# Prevalence of atrophic gastritis in southwest China and predictive strength of serum gastrin-17: A cross-sectional study (SIGES)

**DOI:** 10.1038/s41598-020-61472-7

**Published:** 2020-03-11

**Authors:** Rui Wang, Xin-Zu Chen

**Affiliations:** 1 Department of Gastroenterology, Nursing Section, West China Hospital, Sichuan University, Chengdu, China; 2 Department of Gastrointestinal Surgery & Laboratory of Gastric Cancer, West China Hospital, Sichuan University, Chengdu, China; 3 Department of Gastrointestinal and Hernia Surgery, The Second People’s Hosopital of Yibin • West China Yibin Hospital, Sichuan University, Yibin, China; 4 Department of General Surgery, The First People’s Hospital of Longquanyi • West China Longquan Hospital, Sichuan University, Chengdu, China

**Keywords:** Predictive markers, Gastrointestinal diseases

## Abstract

A hospital-based cross-sectional study in SIGES project was conducted during 2016.5–2017.5 in West China Hospital. It was aimed to observe the prevalence of atrophic gastritis (AG) in southwest China, and assess the diagnostic strength of serum gastrin-17 (G-17) in predicting AG in Chinese population. Asymptomatic healthy controls from health check-up, cancer-free patients with unspecific upper gastrointestinal symptoms, and histologically proven gastric cancer patients were eligible, if serum pepsinogen-I (PG-I), PG-II, and G-17 were detected. AG status was classified by the accredited cutoffs of PG-I (<70 ug/L) and PG-I/II ratio (<3). Totally, healthy controls (n = 9,425), symptomatic patients (n = 671) and gastric cancer patients (n = 305) were simultaneously observed, in which the prevalence of AG in southwest China were estimated as 15.9/1,000, 28.3/1,000, and 55.7/1,000 persons, respectively. The age-specific prevalence of AG in healthy controls showed a significantly uphill trend (p for trend <0.001). Higher level of serum G-17 was significantly associated with increased risk of AG in healthy population (15–30 pmol/L, aOR = 20.67, 95% CI 9.17–46.55; >30 pmol/L, aOR = 314.41, 95% CI 166.10–595.12). Throughout the progression of stomach diseases, the diagnostic strength of serum G-17 for AG showed a downhill trend across more advanced situations. In despite of that, serum G-17 displayed a good performance in predicting AG in the entire cross-sectional population (AUC = 0.92, 95% CI 0.89–0.94; SEN = 85.5%; SPE = 93.2%; LR+ = 12.55; LR− = 0.11). Population in southwest China had intermediate prevalence of AG, while the prevalence was increased over age or disease progression. High level of serum G-17 might be a reliable non-invasive measurement to predict AG in southwest Chinese population.

## Introduction

Atrophic gastritis is a well-established precursor of intestinal-type gastric cancer. The population prevalence of atrophic gastritis generally ranged from 2.1% to 8.2%^1^. The presence of atrophic gastritis was associated with the increased risk of gastric cancer during longitudinal observation in both the western and eastern populations^2–4^. Therefore, the China Consensus on Early Gastric Cancer Screening, Endoscopic Diagnosis and Treatment (2014) recommended to define patients with atrophic gastritis as a kind of high-risk candidates for gastric cancer screening^5^. The European Helicobacter Study Group agreed on the statement “Serologic screening is suitable for clinical use in countries with a relatively low incidence of gastric cancer, because it enables endoscopic follow-up of caseswith an abnormal serologic profile suggesting atrophic gastritis”^6^. However, nationwide massive screening programs for gastric cancer were established only in Japan and Korea, but not in China and other nations yet^7,8^. Regarding the great health burden from gastric cancer in China^9,10^, particularly the relatively low proportion of early gastric cancer^7,11^, it would be necessary to investigate the relevant screening strategy covering precancerous lesions based on Chinese population.

Serum pepsinogen was widely used in clinical practice as biomarkers of stomach inflammation and mucosal lesions, including atrophic changes^12,13^. Serologic diagnosis of atrophic gastritis by combination of pepsinogen-I (PG-I) and PG-I/II ratio demonstrated excellent performance as a preferable non-invasive measurement^14–16^. Particularly, pepsinogens was recommended as useful biomarkers to reflect gastric atrophy and identify high-risk subpopulation for gastric cancer by an Asia-Pacific consensus^17^. Additionally, gastrin-17 (G-17) was known as another non-invasive biomarker for atrophic gastritis^18–20^. G-17, a predominant form of antral hormone gastrins in plasma or tissue in antral mucosa, can regulate gastric acid secretion and growth of the gastric mucosa^18,21^. G-17 is almost exclusively produced by the antral G-cells, which can reflect the progression and severity of gastric mucosal diseases. Previous observational studies showed diverse results, and a meta-analysis found the diagnostic strength of G-17 for predicting atrophic gastritis was only 48% sensitivity and 79% specificity^22^. However, the relevant studies based on Chinese population were still sparse, and the extrapolation of serum G-17 test in Chinese massive screening requires further investigations.

Therefore, the SIGES project was aimed to investigate the prevalence of atrophic gastritis in southwest China, and simultaneously assess the diagnostic strength of serum G-17 in predicting atrophic gastritis in healthy persons, symptomatic cancer-free patients, and gastric cancer patients.

## Methods

### Study design

**Figure 1:**
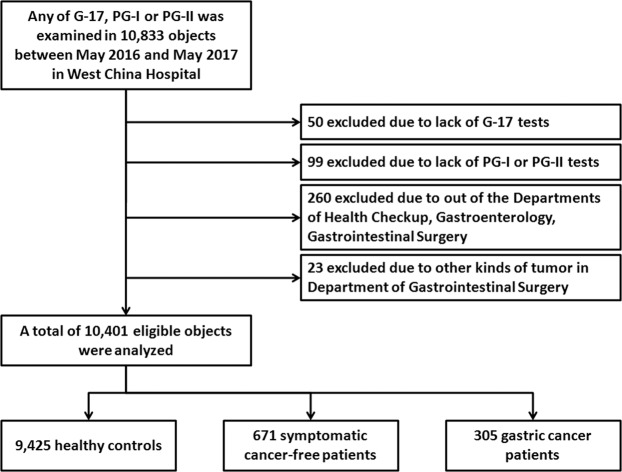
The flow chart of the SIGES cross-sectional study.

This was a retrospective hospital-based cross-sectional study, with the major mission of **Si**chuan **G**astric Cancer **E**arly Detection and **S**creening (SIGES) project. It was conducted in West China Hospital, Sichuan University, a central high-volume teaching hospital at Sichuan province in the southwest of China. The period of the study was between May 2016 and May 2017. The subjects covered healthy controls, symptomatic cancer-free patients, and gastric cancer patients, who were managed or treated in West China Hospital. A total of 10,401 observations were included in this SIGES study (Fig. 1).

### Ethics

This hospital-based cross-sectional study retrospectively collected the participants' basic information and results of serum G-17, PG-I, and PG-II. The SIGES study was approved by the Biomedical Ethical Committee of West China Hospital, Sichuan University (id: 2015-151-V2). The informed consent was waived by the approval of the Biomedical Ethical Committee for the sake of retrospective nature, but the personal information was anonymised, when analyzing and reporting data.

### Eligibility

The eligibility was reviewed and assessed thoroughly based on the electronic medical records reportedby general practioners, gastroenterologists, or gastrointestinal surgical oncologists: (1) The healthy controls were collected from the health checkup in the Center of Health Checkup. The general practitioners recorded them as the asymptomatic status and cancer-free status; (2) The symptomatic cancer-free patients were collected among the outpatients or inpatients from the Department of Gastroenterology and the Department of Gastrointestinal Surgery. These patients were diagnosed of chronic gastritis or functional dyspepsia due to unspecific upper gastrointestinal symptoms, and were recorded as cancer-free status by gastroenterologists or gastrointestinal surgical oncologists; (3) The gastric cancer patients were collected from the Department of Gastrointestinal Surgery. The diagnosis was proved by endoscopic biopsy and pathology, regardless of stage. Other kinds of malignancies were excluded, such as lymphoma and gastrointestinal stromal tumor. In addition, all the included observations should be tested the serum G-17, PG-I, and PG-II together. For the gastric cancer patients, the serologic tests should be performed preoperatively, while the postoperative tests would not be considered.

### Serology

Fasting blood samples were obtained from all observations at health checkup, outpatient visit, or surgical ward. The fresh sample was transferred to the Department of Clinical Laboratory in time and the serum was tested in the same day. The serologic tests of G-17, PG-I, and PG-II were in-house determined by enzyme-linked immunosorbent assay (ELISA; BioHit) according to the manufacturer’s instructions. The in-house cutoffs of normal references were, PG-I 70–165 ug/L, PG-II 3–15 ug/L, PG-I/II ratio 7–20, and G-17 1–15 pmol/L, respectively. The atrophic gastritis was defined as PG-I < 70.0 ug/L and PG-I/II ratio < 3.0, which was a comprehensively accepted cutoff for both the western and eastern populations^3,13,23,24^. Among those, if PG-I < 20.0 ug/L and PG-I/II ratio < 3.0, they were defined as severe atrophy, while the others were mild-moderate atrophy.

### Statistics

Demographic characteristics and serologic results of the observations were compared by Chi-square test for categorical variables, and Wilcoxon rank-sum test for ranked variables or continuous variables without normality distribution. Shapiro-Wilk test for normal data was used to check the normality of variables. Provided multi-group comparison, the Kruskal-Wallis rank test was performed initially. Spearman rho correlation test was used analyzed the association of G-17 with PG-I, PG-II, and PG-I/II ratio. The correlation coefficients were estimated, and relationship was classified by the absolute value of coefficients, as strong (0.7–1), moderate (0.5–0.7), weak (0.3–0.5), and none (0–0.3). The non-parametric trend test was used to test the changes of prevalence over age groups. Multivariate analyses were performed by Logistic regression, and adjusted odds ratio (aORs) with 95% confidence intervals (CIs) were estimated. Non-parametric ROC analysis without covariates was used to assess the capability of predicting atrophic gastritis status, while sensitivity (SEN), specificity (SPE), accurancy (ACC), positive likelihood ratio (LR+), and negative likelihood ratio (LR-) were additionally estimated. The LR+ > 10.0 or LR− < 0.1 presented a strong diagnostic power. The area under the curve (AUC) with standard error (SE) was calculated. The cutoffs of serum G-17 > 1 pmol/L, >15 pmol/L, and > 30 pmol/L were applied while estimating AUCs. Additionally, the maximal Youden index (=SEN + SPE) was calculated to determine the optimal cutoff of serum G-17. The strength of AUCs was classified as mild (0.5–0.7), moderate (0.7–0.9), and strong (0.9–1). The difference of AUCs were compared by the Z test. All statistical tests were two-sided and statistical significance was defined as p < 0.05. The STATA/SE 12.0 software was used for statistical analysis.

## Results

In the SIGES study, 9,425 healthy controls, 671 symptomatic cancer-free patients, and 305 gastric cancer patients were included. The demography and results of serum PG-I, PG-II, PG-I/II ratio, and G-17 were displayed in the Table 1 and Supplementary Fig. 1. There was only weak-moderate correlation of serum G-17 with serum PG-I, PG-II, or PG-I/II ratio (Supplementary Fig. 2).

**Table 1:** Demography and tests of pepsinogens and gastrin-17 at the baseline.

	Healthy controls (n = 9,425)	Symptomatic cancer-free patients (n = 671)	Gastric cancer patients (n = 305)	p_1_	p_2_
**Sex**					
Males	5,087 (54.0%)	304 (45.3%)	208 (68.2%)	<0.001	<0.001
Females	4,338 (46.0%)	367 (54.7%)	97 (31.8%)		
**Age (year)**					
<20	21 (0.2%)	1 (0.2%)	0	<0.001	<0.001
20–39	2,372 (25.2%)	110 (16.4%)	27 (8.9%)		
40–59	5,781 (61.3%)	371 (55.3%)	121 (39.7%)		
60–79	1,210 (12.8%)	185 (27.6%)	149 (48.9%)		
80–	41 (0.4%)	4 (0.6%)	8 (2.6%)		
**PG-I (ug/L)**					
Normal (70–165)	5,338 (56.6%)	375 (55.9%)	142 (46.6%)	<0.001	<0.001
Very low (<20)	99 (1.1%)	11 (1.6%)	6 (2.0%)		
Low (20, <70)	3,657 (38.8%)	155 (23.1%)	49 (16.1%)		
High (>165)	331 (3.5%)	130 (19.4%)	108 (35.4%)		
**PG-II (ug/L)**					
Normal (3–15)	7,633 (81.0%)	466 (69.4%)	149 (48.8%)	<0.001	<0.001
Low (<3)	189 (2.0%)	16 (2.4%)	2 (0.7%)		
High (>15)	1,603 (17.0%)	189 (28.2%)	154 (50.5%)		
**PG-I/II ratio**					
Normal (7–20)	7,247 (76.9%)	514 (76.6%)	168 (55.1%)	0.028	<0.001
Very low (<3)	214 (2.3%)	22 (3.3%)	23 (7.5%)		
Low (3, <7)	1,827 (19.4%)	109 (16.2%)	104 (34.1%)		
High (>20)	137 (1.5%)	26 (3.9%)	10 (3.3%)		
**G-17 (pmol/L)**					
Normal (1–15)	5,885 (62.4%)	380 (56.6%)	187 (61.3%)	<0.001	<0.001
Low (<1)	2,904 (30.8%)	162 (24.1%)	28 (9.2%)		
High (>15, 30)	334 (3.5%)	73 (10.9%)	49 (16.1%)		
Very high (>30)	302 (3.2%)	56 (8.4%)	41 (13.4%)		

Abbreviations: G-17, gastrin-17; PG-I, pepsinogen-I; PG-II, pepsinogen-II.

p_1_, for comparisons between symptomatic cancer-free patients and healthy controls.

p_2_, for comparisons between gastric cancer patients and healthy controls.

**Figure 2:**
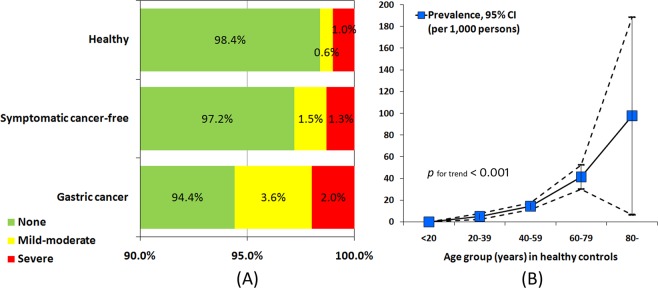
(**A**) The proportions of atrophic gastritis by serologic classification in different subjects, and (**B**) the age-specific prevalence (per 1,000 persons) in healthy controls.

The prevalence of atrophic gastritis were 15.9 (95% CI 13.4–18.4) per 1,000 persons in healthy population, 28.3 (95% CI 15.8–40.9) per 1,000 persons in upper GI symptomatic patients, and 55.7 (95% CI 30.0–81.5) per 1,000 persons in gastric cancer patients, respectively (Fig. 2). In the healthy controls, the age-specific prevalence of atrophic gastritis showed a significantly uphill trend (p for trend < 0.001) (Fig. 2). The prevalence were 0, 5.1 (95% CI 2.2–7.9), 14.5 (95% CI 11.4–17.6), 41.3 (95% CI 30.1–52.5), and 97.6 (95% CI 6.7–188.4) per 1,000 persons in <20 years, 20–39 years, 40–59 years, 60–79 years, and ≥80 years age groups, respectively.

**Figure 3:**
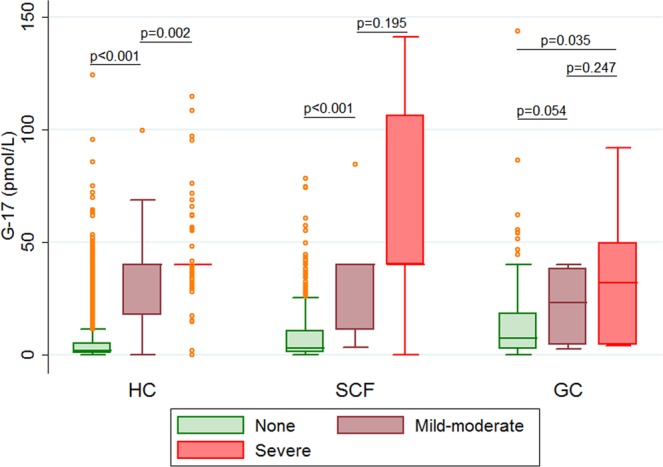
Box plots of serum G-17 levels by severity of atrophic gastritis in healthy controls (HC), symptomatic cancer-free patients (SCF), and gastric cancer patients (GC).

Sex was not associated with the risk of atrophic gastritis (Table 2). Concerning age, only ≥60 years was an independent risk factor in healthy population (aOR = 3.02, 95% CI 1.46–6.21). The serum G-17 level presented uphill trends over the severity of atrophic gastritis in different subjects (all p for trend < 0.05) (Fig. 3). Additionally, in healthy controls, high level (aOR = 20.67, 95% CI 9.17–46.55) and very high level (aOR = 314.41, 95% CI 166.10–595.12) of serum G-17 were associated with the increased risks of atrophic gastritis. However, low level of serum G-17 was not associated with the risk of atrophic gastritis (Table 2), and it was the reason why low and normal levels of serum G-17 were combined as one subgroup in the present study. Moreover, throughout the progression of stomach diseases, only very high level of serum G-17 was always associated with the increased risk of atrophic gastritis in symptomatic cancer-free patients (aOR = 42.40, 95% CI 11.63–154.66) and gastric cancer patients (aOR = 5.50, 95% CI 1.78–17.02), respectively.

**Table 2:** The risks of atrophy gastritis in groups of different subjects.

Covariate	Healthy controls		Symptomatic cancer-free		Gastric cancer	
	CAG prevalence	aOR (95% CI)	CAG prevalence	aOR (95% CI)	CAG prevalence	aOR (95% CI)
**Sex**						
Female	17.1‰	ref.	38.1‰	ref.	72.2‰	ref.
Male	14.9‰	1.00 (0.68–1.49)	16.4‰	0.44 (0.14–1.33)	48.1‰	0.71 (0.25–2.05)
**Age (yrs)**						
<40	5.0‰	ref.	0‰	—	0‰	—
40–59	14.5‰	1.82 (0.93–3.58)	32.3‰	ref.	66.1‰	ref.
60–	43.2‰	3.01 (1.46–6.21)	37.0‰	1.20 (0.42–3.44)	57.3‰	1.43 (0.50–4.10)
**Serum G-17**						
Normal	1.9‰	ref.	7.9‰	ref.	37.4‰	ref.
Low	1.7‰	0.96 (0.33–2.76)	6.2‰	0.87 (0.09–8.53)	0‰	—
High	38.9‰	20.67 (9.17–46.55)	13.7‰	1.97 (0.20–19.31)	61.2‰	1.71 (0.42–6.98)
Very high	400.7‰	314.41 (166.10–595.12)	250.0‰	42.40 (11.63–154.66)	170.7‰	5.50 (1.78–17.02)

Abbreviations: aOR, adjusted odds ratio; CAG, chronic atrophic gastritis; CI, confidence interval; G-17, gastrin-17; ref., reference.

**Figure 4:**
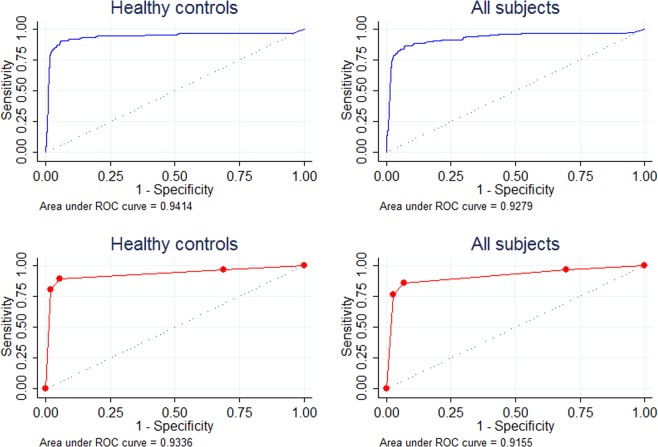
The ROCs of serum G-17 predicting atrophic gastritis in different subjects. (blue lines for continuous variable; red lines for categorical variable by in-house cutoffs).

Regarding none inverse risk of atrophic gastritis between low and normal levels of serum G-17, the optimal cutoff of prediction would be reasonable. The in-house upper limit of serum G-17 (≤15 pmol/L) demonstrated a good diagnostic performance, with SEN = 89.3%, SPE = 94.6%, ACC = 94.5%, LR+ = 16.51, LR− = 0.11, and AUC = 0.93 (95% CI 0.91–0.96) (Table 3). The optimal cutoff of G-17 by the maximal Youden index was 14.62 pmol/L, which was fairly close to the in-house upper limit and displayed similar diagnostic strength (AUC = 0.94, 95% CI 0.91–0.97, p = 0.294). Throughout the progression of stomach diseases, the diagnostic strength of serum G-17 for atrophic gastritis showed a downhill trend across symptomatic cancer-free patients (AUC = 0.85, p = 0.142) and gastric cancer patients (AUC = 0.70, p<0.001). Nevertheless, in the entire cross-sectional population, the diagnostic strength was still preserved well (AUC = 0.92, p = 0.648) (Fig. 4).

**Table 3:** The diagnostic capability of serum G-17 for AG status in different subjects.

G-17 cutoffs(pmol/L)	SEN	SPE	ACC	LR+	LR−	AUC (95% CI)	p
**Healthy**						0.93 (0.91–0.96)	ref.
>1	96.7%	31.3%	32.3%	1.41	0.11		
>15	89.3%	94.6%	94.5%	16.51	0.11		
>30	80.7%	98.1%	97.8%	41.34	0.20		
**Healthy (continuous variable of G-17)**						0.94 (0.91–0.97)	0.294
≥14.62 (Youden max)	90.7%	94.3%	94.2%	15.87	0.10		
**Symptomatic cancer-free**						0.85 (0.74–0.96)	0.142
>1	94.7%	24.7%	26.7%	1.26	0.21		
>15	79.0%	82.5%	82.4%	4.52	0.26		
>30	73.7%	93.6%	93.0%	11.44	0.28		
**Gastric cancer**						0.70 (0.57–0.82)	<0.001
>1	100%	9.7%	14.8%	1.11	0		
>15	58.8%	72.2%	71.5%	2.12	0.57		
>30	41.2%	88.2%	85.6%	3.49	0.67		
**All observations**						0.92 (0.89–0.94)	0.648
>1	96.8%	30.2%	31.4%	1.39	0.11		
>15	85.5%	93.2%	93.1%	12.55	0.16		
>30	76.3%	97.5%	97.1%	30.34	0.24		

Abbreviations: ACC, accurancy; AUC, area under the curve; CI, confidence interval; G-17, gastrin-17; LR+, positive likelihood ratio; LR−, negative likelihood ratio; ref., reference; SEN, sensitivity; SPE, specificity.

## Discussion

In this cross-sectional study, symptomatic patients and gastric cancer patients in addition to healthy population were simultaneously observed. The prevalence of atrophic gastritis in southwest China were estimated as 15.9/1,000 in healthy population, 28.3/1,000 in upper GI symptomatic patients, and 55.7/1,000 in gastric cancer patients, respectively. High level of serum G-17 (>15 pmol/L) was significantly associated with increased risk of atrophic gastritis in healthy population. Throughout the progression of stomach diseases, the diagnostic strength of serum G-17 for atrophic gastritis showed a downhill trend across more advanced situations. In despite of that, serum G-17 displayed a good performance in predicting atrophic gastritis in entire cross-sectional population.

The prevalence of atrophic gastritis was diverse in different populations in the world. In a Russian asymptomatic cohort, the prevalence of atrophic gastritis was much higher as 108/1,000 persons, while the prevalence of Helicobacter pylori (H. pylori) infection was as high as 76.7% in the cohort^25^. Similarly, in a Kazakhstan cohort (both asymptomatic and symptomatic), the prevalence of atrophic gastritis was up to 141/1,000 persons, with H. pylori infection infection (but no atrophy) in 62.3% of the cohort^26^. In a Cameroon small-sized dyspeptic series, the prevalence of atrophic gastritis was 66/1,000 persons, and the rate of H. pylori infection was 79.8%^27^. In Korea, one of the high-incidence nations of gastric cancer, a multicenter study found the prevalence of endoscopic atrophic gastritis was even up to 407/1,000 persons, and H. pylori seropositivity was a risk factor for intestinal metaplasia, a more advanced precancerous lesion^28^. Additionally, a Swedish long-term cross-sectional study revealed the prevalence of atrophic gastritis was associated with the H. pylori seropositivity^29^.

The information on the prevalence of atrophic gastritis was scarce in Chinese. In contrast, the healthy and symptomatic subjects in the SIGES study appeared only 15.9/1,000 and 28.3/1,000 persons had atrophic gastritis. Among the elder healthy persons, the prevalence were 41.3/1,000 and 97.6/1,000 persons in the age groups 60–79 years and ≥80 years, respectively. The lower prevalence in southwest China may attribute to the decreasing rate of H. pylori infection. Although China was a high-incidence nation of gastric cancer, the prevalence of H. pylori infection displayed a apparent geographic disparity^9^. In a longitudinal observation based on sequential cross-sectional studies in Sichuan, the rate of H. pylori infection declined dramatically from 56.6% to 41.1% between 2008 and 2014^30^. Therefore, it can be inferred that the efforts in health education, screening and control of H. pylori in Sichuan province during the past decade makes the H. pylori-associated atrophic gastritis decreased. A study in two cities of Fujian province indicated the higher prevalence of H. pylori and atrophic gastritis might result in a higher risk condition for developing gastric cancer and a greater population mortality rate^31^. Therefore, these evidence based on Chinese population validated the value of massive screening of atrophic gastritis for the sake of gastric cancer control in China.

In the western populations, it was found the combination of serum PG-I, PG-II, G-17, and H. pylori antibody could obtain acceptable sensitivity and specificity to predict atrophic gastritis^32^. In a multicenter study, the combination had good agreement with endoscopic and biopsy findings in gastric histology of atrophy^33^. However, in contrast, another multicenter prospective study found the combination was not accurate enough to predict atrophic gastritis among dyspeptic patients with sensitivity 50% and specificity 80%^34^. Due to inconsistent results, a meta-analysis pooled twenty diagnostic studies on the combination, and found the summarized sensitivity was 74.7% (95% CI 62.0%-84.3%) and specificity was 95.6% (95% CI 92.6%-97.4%) for diagnosis of atrophic gastritis^35^. Another meta-analysis found the combination performed even better in the corpus atrophy than in the antrum atrophy^36^. Likewise, a cross-sectional analysis based on a northeast Chinese population (12,112 subjects) also proved the usefulness of the combination in identifying high-risk individuals for further diagnostic gastroscopy, and stratifying individuals' risk^24^. A recent updated meta-analysis reported the pooled sensitivity was 0.59 (95% CI 0.38–0.78), while on the other hand, the pooled specificity, diagnostic odds ratio, and AUC were 0.89 (95% CI 0.70–0.97), 12 (95% CI 6–25), and 0.81 (95% CI 0.77–0.84), respectively^13^.

In fact, there was no efficient biomarkers to directly diagnose precancerous lesions of gastric cancer till now^37,38^. However, in the pattern of massive screening, the application of serum pepsinogen assay may be reasonable as a primary classification standard. The present study was aimed to validate the predicting strength of serum G-17 in a massive setting. In the future, we plan to validate the combination of both pepsinogens and G-17 to predict atrophic gastritis in a histology-based setting, but in a smaller group of observations. Additionally, the combination of detecting H. pylori, Epstein-Barr virus, and other oncoviruses might be considerable to further investigate the predicting strength of precancerous lesions in a non-invasive pattern^30,39–41^. Therefore, the above findings warrant further investigation based the SIGES population to identify high-risk subpopulation with precancerous condition for endoscopic screening in a cost-effective manner.

Edkins JS first reported gastrin functioning in the chemical mechanism of gastric acid secretion^42^. The expression of gastrin can also be adjusted by the acid feedback^43^. Among the family of gastrin, G-17 particularly plays a critical role in the regulation of gastric acid secretion^43^. Some other researches similarly found high level of serum gastrin was associated with precancerous lesions and gastric cancer in Asians^44–47^. The results of the present study demonstrated that the prevalence of atrophic gastritis tended to increase from benign to malignant lesion. Atrophy might make acid secretion decreased, while the serum G-17 would be increased through acid feedback adjustment. Similarly, the proton pump inhibitor could make the acid secretion inhibited, and led to the elevated level of serum G-17^48^. It may be the mechanism why a high level of serum G-17 is potentially able to predict precancerous lesions of gastric cancer.

There were some limitations need consider with caution. First, this was a hospital-based cross-sectional population, rather than a natural population. There might be a certain sampling bias. Second, the classification of atrophic gastritis was dependent on serologic measurement, rather than endoscopy and histology. A minor part of misclassification might not be eliminated in this study. The upper GI endoscopy was not mandatory in the massive health checkup in China, so it was impossible to be improved in a cross-sectional study. The serologic classification by the combination of serum PG-I and PG-I/II ratio could achieve a good diagnostic accurancy, even as high as more than 90%^1,23^. However, the updated meta-analysis found the pooled sensitivity of serum pepsinogen assay was merely 59% to predict atrophic gastritis^13^. It means the prevalence of atrophic gastritis might be underestimated in the present study. Third, the health checkup did not involve endoscopic screening and confirmation of the absence of gastric cancer. However, the crude incidence of gastric cancer in China was 33.66/100,000 persons^49^, and the underestimated gastric cancer might be rare in the healthy controls. The sensitivity analysis including the entire cross-sectional population found the contamination with gastric cancer patients (305/10,401) did not impair the diagnostic strength of serum G-17 for prewarning of atrophic gastritis. Fourth, there was methodological concerns why low and normal levels of G-17 were combined. The prevalence of atrophic gastritis were quite close between normal and low levels of G-17, while the aORs were not significant in the healthy and symptomatic cancer-free controls, as well as null atrophic gastritis in low level of G-17 in gastric cancer patients (Table 2). Therefore, in the following analysis shown in the Table 3, the serum G-17 was analyzed as a continuous variable rather than a binary variable. Fifth, the administration of the proton pump inhibitor (PPI) was unclear at the baseline, especially in symptomatic and gastric cancer patients. Recent usage of PPI might lead to the elevated level of serum G-17^48^. It might be the reason why only very high level (>30 pmol/L) of G-17 was associated with atrophic gastritis status in symptomatic and gastric cancer patients. Finally, the consideration of the joint assessment along with H. pylori status in the following investigation of SIGES would be more informative to understand the high-risk subpopulation in southwest China for health policy decision-maker.

In conclusion, population in southwest China had intermediate prevalence of AG, while the prevalence was increased over age or disease progression. In the SIGES study, serum G-17 was found to be a reliable non-invasive measurement to predict AG in Chinese population. However, the decision-making based on serum G-17 in massive screening need further investigate in prospective epidemiologic studies.

## Supplementary information

Supplementary figure 1.

Supplementary figure 2.
