# Supplementary material for: Prevalence of atrophic gastritis in southwest China and predictive strength of serum gastrin-17: A cross-sectional study (SIGES)
Source: Sci Rep. 2020 Mar 11;10:4523. doi: 10.1038/s41598-020-61472-7 (PMC7066171; doi:10.1038/s41598-020-61472-7)
Supplement: Supplementary file 2 — Supplementary figure 2. [file 41598_2020_61472_MOESM2_ESM.pdf]

Prevalence of atrophic gastritis in southwest China and predictive strength of serum gastrin-17: A cross-sectional study (SIGES)

Authors: Rui Wang;[1] Xin-Zu Chen[2,3,4]; on the behalf of the SIGES research group

Affiliations:

1. Department of Gastroenterology, Nursing Section, West China Hospital, Sichuan University, Chengdu, China.
2. Department of Gastrointestinal Surgery & Laboratory of Gastric Cancer, West China Hospital, Sichuan University, Chengdu, China.
3. Department of Gastrointestinal and Hernia Surgery, the Second People's Hosopital of Yibin City · West China Yibin Hospital, Sichuan University, Yibin, China.
4. Department of General Surgery, the First People's Hospital of Longquanyi District · West China Longquan Hospital, Sichuan University, Chengdu, China.

Correspondence:

Xin-Zu Chen, Department of Gastrointestinal Surgery, West China Hospital, Sichuan University, Guo Xue Xiang 37, Chengdu 610041, Sichuan, China. Email: chenxinzu@scu.edu.cn

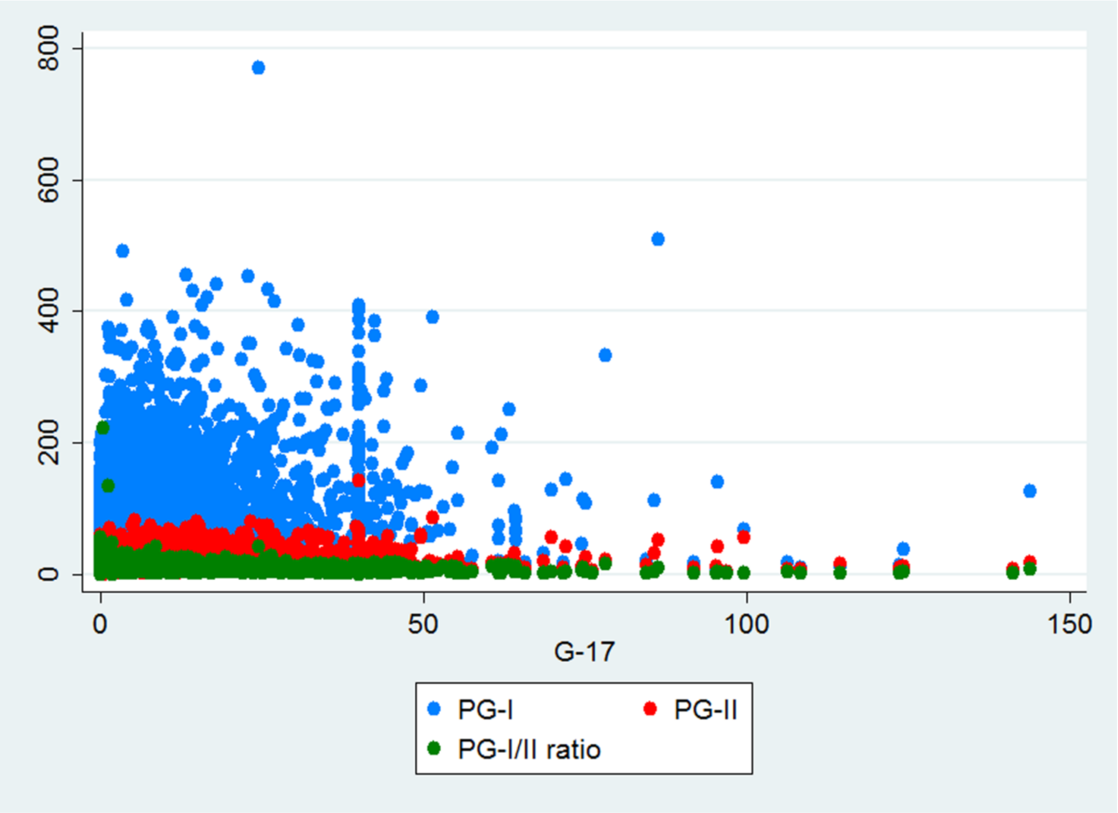

	PG-I		PG-II		PG-I/II ratio	
	Coefficient	p	Coefficient	p	Coefficient	p
Both sexes	0.29	<0.001	0.50	<0.001	-0.44	<0.001
Males	0.28	<0.001	0.50	<0.001	-0.44	<0.001
Females	0.35	<0.001	0.53	<0.001	-0.45	<0.001

Supplementary figure 2. The weak-moderate correlation of serum G-17 with PG-I, PG-II, and PG-I/II ratio.
